# The Quality of Colonoscopy Reporting in Usual Practice: Are Endoscopists Reporting Key Data Elements?

**DOI:** 10.1155/2016/1929361

**Published:** 2016-08-07

**Authors:** S. D. Hadlock, N. Liu, M. Bernstein, M. Gould, L. Rabeneck, A. Ruco, R. Sutradhar, J. M. Tinmouth

**Affiliations:** ^1^Montfort Hospital, Gastroenterology, Ottawa, ON, Canada K1K 0T2; ^2^Institute for Clinical Evaluative Sciences, Toronto, ON, Canada M4N 3M5; ^3^Sunnybrook Health Sciences Centre, Toronto, ON, Canada M4N 3M5; ^4^Toronto Digestive Disease Associates Inc, Toronto, ON, Canada L4L 4Y7; ^5^Cancer Care Ontario, Toronto, ON, Canada M5G 2L7; ^6^Department of Medicine, University of Toronto, Toronto, ON, Canada M5S 1A8; ^7^Institute of Health Policy, Management and Evaluation, University of Toronto, Toronto, ON, Canada M5T 3M6; ^8^Dalla Lana School of Public Health, University of Toronto, Toronto, ON, Canada M5T 3M7

## Abstract

*Background*. High quality reporting of endoscopic procedures is critical to the implementation of colonoscopy quality assurance programs.* Objective*. The aim of our research was to (1) determine the quality of colonoscopy (CS) reporting in “usual practice,” (2) identify factors associated with good quality reporting, and (3) compare CS reporting in open-access and non-open-access procedures.* Methods*. 557 CS reports were randomly selected and assigned a score based on the number of mandatory data elements included in the report. Reports documenting greater than 70% of the mandatory data elements were considered to be of good quality. Physician and procedure factors associated with good quality CS reporting were identified.* Results*. Variables that were consistently well documented included date of the procedure (99.6%), procedure indication (88.9%), a description of the most proximal anatomical segment reached (98.6%), and documentation of polyp location (97.8%). Approximately 79.4% of the reports were considered to be of good quality. Gastroenterology specialty, lower annual CS volume, and fewer years in practice were associated with good quality reporting.* Discussion*. CS reporting in usual practice in Ontario lacks quality in several areas. Almost 1 in 5 reports was of poor quality in our study.* Conclusions*. Targeted interventions and/or use of mandatory fields in synoptic reports should be considered to improve CS reporting.

## 1. Introduction

Colonoscopy (CS) is an important tool used to screen for cancerous and precancerous lesions of the colon. With increasing public awareness of the need for colorectal cancer (CRC) screening, the use of CS has been steadily rising [1]. Increasing use of CS, particularly in the context of organized CRC screening programs, has contributed to a growing interest in improving the quality of endoscopic procedures and measuring specific quality indicators [2–4]. Until recently, the lack of standardized accepted approach to CS reporting was a significant obstacle to measuring these quality indicators. Furthermore, the CS report is also the primary medicolegal documentation as it provides a detailed description of the procedure performed [5]. Open-access (OA) procedures, where the patient is seen directly for CS without a recent office visit with the endoscopist performing the procedure, are increasingly being done [6], which may require more extensive documentation to sufficiently capture all pertinent information. Finally, the quality of the CS report may have a significant impact on clinical care as inadequate documentation may result in needlessly repeating procedures, exposing patients to unnecessary risks.

In 1999, the American Society for Gastrointestinal Endoscopy published guidelines for quality improvement of endoscopy [7]. More recently, the Quality Assurance Task Force of the National Colorectal Cancer Roundtable (NCCRT) has developed a data reporting system for colonoscopy (CO-RADS), which outlines the essential elements to include in each CS report and highlights key indicators for quality improvement [8]. However, despite the recent interest in measuring the quality of CS reporting, only a limited number of studies have addressed this important issue [9–20]. Most prior work has been limited to single academic institutions and/or certain populations [9, 11, 13–20], computer generated reporting [10, 11, 13, 15, 16, 19], or intervention and/or audit studies [13, 16–20]. These factors have limited the generalizability of prior studies. The aims of our research were to (1) determine the quality of CS reporting in clinical practice using established CS reporting guidelines; (2) identify factors associated with good quality CS reporting; and (3) compare CS reporting in OA and non-open-access (NOA) procedures.

## 2. Methods 

### 2.1. Data Sources

This study evaluated CS reports that were obtained from a larger parent study that was designed to validate colonoscopy data elements in Ontario's health administrative databases using a large scale population-based chart review. These datasets, held at the Institute for Clinical Evaluative Sciences (ICES) and the Canadian Institute for Health Information (CIHI), are linked using unique, encoded identifiers and analyzed at ICES. Using a stratified sampling strategy, the parent study randomly selected 1,400 CS procedures from fiscal year (FY) 2008 from 30 randomly selected facilities (24 hospitals and 6 nonhospital endoscopy clinics) in Ontario using the Ontario Health Insurance Plan (OHIP) Claims History Database, the National Ambulatory Care Reporting System (NACRS), and the CIHI-Discharge Abstract Database (DAD).

From the larger parent study, a convenience sample of 575 reports was randomly selected for analysis in the current study as reabstraction of reports was required to evaluate quality of CS reporting. Report formats ranged from hand-written to dictated notes to proprietary or local software with or without electronic medical record (EMR). All personal health information, including endoscopist name, were removed from the reports prior to review. Each report was identified by date of procedure and the ICES key number (IKN), which is an encrypted version of the provincial health insurance plan number. Using the IKN, the data abstracted from each report were linked to the Ontario health administrative databases in order to analyze physician and procedure factors associated with good CS reporting.

Ethics approval for this study was obtained from the Sunnybrook Health Sciences Centre Research Ethics Board.

### 2.2. Evaluation of CS Report Quality

We convened a panel of endoscopists with expertise on the topic (JT, SH, LR, MG, and MB) prior to evaluating the CS reports. The objective of the panel was to decide on the mandatory quality indicators required for good quality CS reporting. The panel reviewed the 1999 ASGE guidelines [7] and the 2007 NCCRT recommendations for CS reporting [8]. All data elements recommended by these reports (preprocedure, procedure, and postprocedure) as necessary for high quality CS reporting were reviewed by the panel. The panel then identified 33 data elements to be used to evaluate the quality of CS reporting in our sample based on existing guidelines. Of these, the panel categorized 13 data elements as “mandatory” (Table 1) based on their perceived importance. These include indication and date of the procedure, sedation name and dose, anatomical segment reached, cecal/terminal ileum (TI) landmarks through written indication, quality of bowel preparation, location and size of any mass found, location and size of any polyp found, summary statement, and management plan. Each CS report was assigned a score based on the number of mandatory data elements included in the report (1 point for each data element). Generally, elements were categorized as nonmandatory if it was likely that they would be captured in the documentation of the preendoscopy consultation visit.

### 2.3. Physician and Procedure Factors Analyzed

Using the databases housed at ICES, we collected information on the institution (teaching/community hospital or private clinic) where the CS was performed, whether it was an OA procedure (patient seen directly for CS without an office visit with the endoscopist performing the procedure in the prior 5 years), and physician factors. Physician factors included specialty (gastroenterology, surgery, internal medicine, and general practitioner/other), number of years in practice (number of years since graduation from medical school), annual CS volume (mean annual number of procedures completed in the preceding year), CS completion rate (proportion of successful cecal/TI intubations in the preceding year), and polypectomy rate (proportion of CS procedures in the preceding year where at least one polyp was detected).

### 2.4. Statistical Analysis

CS reports were evaluated using a standardized data collection form. Reports were assigned a score from 0 to a maximum of 13, with each mandatory data element representing one point. The number and proportion of CS reports complying with each mandatory data element were recorded. Reports documenting ≥70% of the possible mandatory data elements were classified as “good” quality reports and the remaining reports were classified as “poor” quality based on a similar threshold used in previous work [9]. For this calculation, the denominator varied depending on the number of measurable data elements (e.g., if there were no polyps or masses, related data elements were not included in the denominator for that specific report). In analyzing the quality of CS reporting in OA procedures, we also evaluated the documentation of nonmandatory data elements. As OA procedures were not preceded by a consultation visit, it was assumed that reports from these procedures should be more comprehensive than the reports from NOA procedures.

Univariate and multivariable logistic regression were used to model physician and institutional factors (institution; OA status; physician specialty; years in practice; annual CS volume; polypectomy rate; CS completion rate) associated with the binary outcome of good quality CS reporting. A generalized estimating equations approach was implemented to account for clustering of colonoscopies within physicians [21]. All analyses were conducted using SAS v.9 statistical software (SAS Institute, Cary, NC).

## 3. Results

Five hundred and seventy-five CS reports (495 from hospitals and 80 from private clinics) were evaluated. Reports by physicians missing information on annual CS volume were excluded from further analyses (*n* = 18). Colonoscopies were performed by 114 physicians at 15 sites (10 hospitals and 5 private clinics) in Ontario. Each physician and each site contributed from 1 to 25 colonoscopies and from 9 to 113 reports to the sample, respectively.

**Figure 1:**
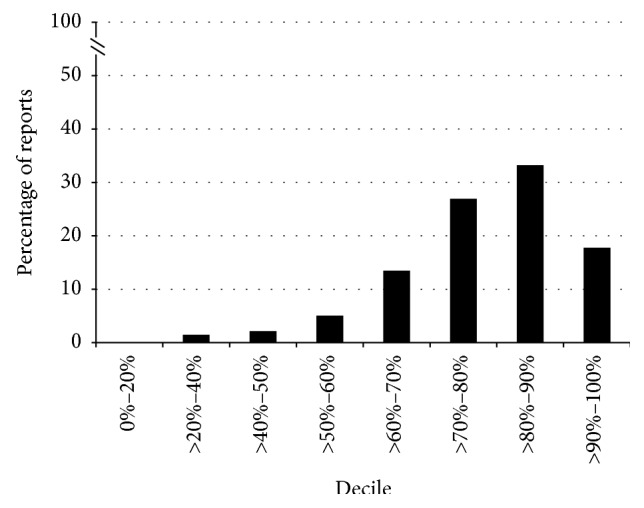
Distribution of the reports by decile of mandatory data elements reported, from 0 to 100%.

Documentation of the mandatory data elements is reported in Table 1. Variables that were consistently well documented included date of the procedure (99.6%), procedure indication (88.9%), a description of the most proximal anatomical segment reached (98.6%), and documentation of polyp location (97.8%). By contrast, the quality of bowel preparation (34.5%) and polyp size (45.9%) were documented in less than half of all reports. Over 20% of reports did not include a summary statement or follow-up plan. When the reports were assigned a score representing the overall completeness of documentation, 442 (79.4%) of the reports were considered good quality (documenting ≥70% of the mandatory indicators).  Figure 1 depicts the distribution of the reports in each decile of completeness, ranging from 0 to 100% of possible mandatory elements. Nonmandatory data elements reported by decile are presented in Supplementary Materials available online at http://dx.doi.org/10.1155/2016/1929361.

Univariate analyses demonstrated that the quality of reporting varied significantly at the physician level for specialty, number of years in practice, CS volume, CS completion rate, and institution type (Table 2). Of the variables included in the multivariate analysis, surgeons and internists were significantly less likely to have good quality reporting compared to gastroenterologists (OR 0.19 (95% CI 0.11–0.34) for surgeons and 0.22 (95% CI 0.09–0.53) for internists) (Table 3). For every additional year in practice, there was a 5% decrease in the odds of good quality reporting. Physicians with higher annual CS volume practices were also less likely to have good quality reporting (OR 0.86 per 100 procedures (95% CI 0.76–0.97)).

Compared to NOA procedures, OA procedures were not associated with good quality of reporting on mandatory data elements in the multivariable analysis. However, several nonmandatory data elements, patient history, family history, comorbidity, and physical exam, were consistently reported to a greater extent in OA procedures compared to NOA procedures (Table 4). Aside from sedation name, which was only documented in 48.0% of OA procedures compared to 70.4% of NOA procedures, OA reports consistently documented all quality indicators to a similar extent or better compared to NOA reports. In general, whether OA or NOA procedure, nonmandatory data elements were documented less well than mandatory elements.

## 4. Discussion

Our study of CS reporting in usual practice in Ontario reveals that 1 in 5 reports fail to document key quality indicators. Good quality CS reporting is associated with gastroenterology specialty, physicians with fewer years in clinical practice, and those with lower CS volumes.

The importance of our study is that it is the first to evaluate the quality of CS reporting in usual clinical practice, given the random sampling method that was used to identify study participants. Previous published studies have been limited in their generalizability for numerous reasons. First, prior work has been limited to single institutions or homogenous populations [9, 11, 13–20]. For example, Palmer et al. [11] reported on the quality of 135 CS reports of community-based physicians but this study was limited to a cohort of patients from a single Veterans Administration (VA) Medical Centre. The homogeneous patient population and restricted geographic variation of the participating physicians limit the applicability of the results to larger population-based practices. Second, CS reports evaluated in previous work have been computer-generated [10, 11, 13, 15, 16, 19], which may not be representative of other jurisdictions [22–25]. Finally, previous reported results have been of audit or intervention studies, where physicians were aware that they were being assessed [13, 16–20].

Prior published studies have reported similar findings to ours. For example, in 2002, Robertson et al. [9] evaluated a single CS report from 122 endoscopy centres in the United States using the 1999 ASGE reporting guidelines as the reference standard. The authors found that the quality of CS reporting was highly variable with indication for the procedure, examination extent, and polyp location and removal being well documented [9]. On the other hand, patient history, informed consent, medications used during the procedure, and adequacy of bowel preparation were poorly documented [9]. The largest study, published by Lieberman et al. [10] in 2009, reviewed 438,521 computer generated reports from the Clinical Outcomes Research Initiative (CORI) database. This study demonstrated higher documentation rates than previously reported but did note considerable variation in the quality of reporting across the 73 practice sites, despite using a standardized computer based reporting tool. More recently, a study examining 4,800 computerized CS reports from 12 endoscopy departments in the Netherlands showed variation among quality indicators with withdrawal time, polyp size, polyp morphology, and quality of bowel preparation being of poorer compliance with reporting among the 117 endoscopists [13]. Singh et al. [12] recently reported on a sample of 797 CS reports from six community and academic hospitals in Manitoba, Canada. Approximately 80% of the reports did not include information on the quality of bowel preparation and only 34% of the reports contained information on polyp morphology [12].

Reporting on the quality of bowel preparation was consistently low (34.5%) in our study and in prior work [9, 12, 13]. One outlier was the study by Beaulieu et al. [15] that examined 250 consecutive computerized endoscopic reports from a tertiary care institution in Quebec, Canada, using the indicators set forth by the Quality Assurance Task Group of the NCCRT. The quality of bowel preparation in this sample was recorded in 99.9% of the reports [15]. The high compliance with reporting in the study by Beaulieu et al. [15] may be a result of using a computerized report generator with compulsory fields, using preformatted text or drop-down menus, or may reflect local practice patterns as all procedures were completed at a single institution. Without a compulsory field for bowel preparation, physicians may assume that they only need to report it when bowel preparation is insufficient.

Several physician factors were associated with good quality CS reporting. Our findings demonstrated higher quality reporting by gastroenterologists and physicians with lower annual CS volumes. One possible explanation is that physicians performing fewer colonoscopies may take more time for procedure documentation. Physicians with fewer years in clinical practice were also found to have better reporting. This finding may reflect the increasing emphasis on clinical documentation and quality assurance in recent medical education or, simply, that, over time, physicians generate less detailed reports that are of poorer quality. Our results identify a number of target groups for quality improvement initiatives; for example, surgical training programs should ensure that residents are aware of the importance and key elements of good endoscopy reporting. Further, individual endoscopy units may also benefit from an audit and feedback tool to assess their CS reporting quality.

Documentation in OA procedures is particularly important as patients are not seen in a prior consultation where information on the patient, medications, and family history would be expected to be documented. However, exploring quality of CS reporting among OA and NOA procedures revealed that several nonmandatory indicators were consistently reported to a greater extent in OA procedures. These included patient history, family history, comorbidity, medication list, and history of previous CS examinations. Although unsurprising as patients attending NOA procedures had been seen in consultation beforehand and nonmandatory elements were likely recorded at that visit, reporting of OA procedures was still suboptimal as a third or more of these reports were missing data elements such as history of presenting illness, comorbidity, and medications.

Our results must be interpreted in consideration of the strengths and limitations of the study. We included data from a large random sample of institutions on CS performed by a large random sample of physicians across Ontario, increasing the generalizability of our findings. Furthermore, our study population was comprised primarily of physicians using nonstandardized CS reporting tools (non-EMR) and is representative of usual practice in many other jurisdictions [25]. Unlike the largest studies limited to EMR/software or single centres, our design captured heterogeneity in usual practice across multiple sites by including various report formats and sources. As others have shown [10, 13], there is important variation even with electronic reporting systems; our results can be used to inform the design of such systems to ensure high quality reports. Limitations include the use of an arbitrary threshold of 70% for determining good quality reporting. However, previous work [9] has also employed a similar threshold. Our findings are not intended to address the quality of the included procedures nor whether included elements were reported correctly; rather our goal was to measure how well endoscopists completed the CS report. The CS report is the most comprehensive description of the procedure and is commonly used to assess the quality of the procedure; reports that are missing key elements will undermine quality improvement initiatives. Lastly, the study does rely in part on accurate entry of billing codes by physicians; however this information was used only to determine which procedure and physician factors were associated with good quality reporting and not to assess the quality of the reports themselves.

In conclusion, CS reporting in usual practice in Ontario lacks quality in several areas. Almost 1 in 5 reports were of poor quality in our study, and bowel prep quality and polyp size were documented in less than half of reports. Targeted interventions and/or use of mandatory fields in synoptic reports should be considered to improve CS reporting.

## Supplementary Material

Supplementary Figure 1 includes the distribution of non-mandatory data elements reported by decile.

## Figures and Tables

**Table 1:** Number and proportion of mandatory data elements documented among CS reports (*N* = 557).

Mandatory data element	Documented, *n* (%)	Total number of reports
*Preprocedure elements*		
Indication	495 (88.9)	557

*Procedure elements*		
Date of procedure	555 (99.6)	557
Sedation: name	364 (65.4)	557
Sedation: dose	318 (57.1)	557
Anatomical segment reached	549 (98.6)	557
Cecal/TI landmarks	287 (67.4)	426
Quality of bowel preparation	192 (34.5)	557
Colonic mass: location	16 (100)	16
Colonic mass: size	11 (68.8)	16
Colonic polyp (1st): location	177 (97.8)	181
Colonic polyp (1st): size	83 (45.9)	181

*Postprocedure elements*		
Summary statement	425 (76.3)	557
Management plan	438 (78.6)	557

CS: colonoscopy; TI: terminal ileum.

**Table 2:** Physician and institution factors associated with good quality CS reporting.

Variable	Value	Good quality (*n* = 442)	Poor quality (*n* = 115)	Total (*N* = 557)	*P* value
Completion rate, *n* (%)	<90%	162 (36.7)	38 (33.0)	200 (35.9)	0.006
	≥90%	280 (63.3)	77 (67.0)	357 (64.1)	

Open-access CS, *n* (%)	No	94 (21.3)	31 (27.0)	125 (22.4)	NS
	Yes	348 (78.7)	84 (73.0)	432 (77.6)	

Polypectomy rate	Mean ± SD	0.22 ± 0.10	0.21 ± 0.09	0.22 ± 0.09	NS
	Median (IQR)	0.20 (0.14–0.28)	0.20 (0.16–0.24)	0.20 (0.15–0.27)	

Annual CS volume	Mean ± SD	429.17 ± 254.25	578.97 ± 372.99	460.10 ± 288.93	<0.001
	Median (IQR)	443 (201–557)	535 (280–752)	465 (202–588)	

Institution type, *n* (%)	Clinic	51 (11.5)	25 (21.7)	76 (13.6)	0.005
	Hospital	391 (88.5)	90 (78.3)	491 (88.2)	

Physician specialty, *n* (%)	Surgeon	205 (46.4)	75 (65.2)	280 (50.3)	<0.001
	Internist	34 (7.7)	17 (14.8)	51 (9.2)	
	GI	203 (45.9)	23 (20.0)	226 (40.6)	

Years in practice	Mean ± SD	23.10 ± 12.00	28.85 ± 11.28	24.28 ± 12.07	<0.001
	Median (IQR)	21 (14–30)	28 (20–39)	23 (15–31)	

CS: colonoscopy; GI: gastroenterologist.

**Table 3:** Multivariable analysis on factors associated with good quality CS reporting.

Variable	OR (95% CI)	*P* value
*Completion rate, *%		
<90%	0.92 (0.56–1.52)	NS
≥90%	1.0	

*Open-access CS*		
No	1.12 (0.64–1.96)	NS
Yes	1.0	

*Polypectomy rate, *%		
Per 1%	0.42 (0.03–5.17)	NS

*Annual CS volume*		
Per 100 procedures	0.86 (0.76–0.97)	0.01

*Institution type*		
Clinic	1.36 (0.46–3.99)	NS
Hospital	1.0	

*Physician specialty*		
Surgeon	0.19 (0.11–0.34)	<0.0001
Internist	0.22 (0.09–0.53)	0.0008
GI	1.0	

*Years in practice, years*		
Per year	0.95 (0.93–0.97)	<0.0001

CS: colonoscopy; GI: gastroenterologist.

**Table 4:** Number and proportion of selected^*∗*^ nonmandatory data elements documented among CS reports by open-access (OA) status.

Variable	Value	Open access (OA) (*n* = 125)	Nonopen access (NOA) (*n* = 432)	Total (*N* = 557)
History, *n* (%)	Yes	79 (63.2)	99 (22.9)	178 (32.0)
	No	46 (36.8)	333 (77.1)	379 (68.0)

Comorbidity, *n* (%)	Yes	75 (60.0)	60 (13.9)	135 (24.2)
	No	50 (40.0)	372 (86.1)	422 (75.8)

Physical exam, *n* (%)	Yes	57 (45.6)	67 (15.5)	124 (22.3)
	No	68 (54.4)	365 (84.5)	433 (77.7)

Medication list, *n* (%)	Yes	66 (52.8)	45 (10.4)	111 (19.9)
	No	59 (47.2)	387 (89.6)	446 (80.1)

Informed consent,^a^ *n* (%)	Yes	58 (46.4)	93 (21.5)	151 (27.1)
	No	67 (53.6)	339 (78.5)	406 (72.9)

Family history, *n* (%)	Yes	76 (60.8)	74 (17.1)	150 (26.9)
	No	49 (39.2)	358 (82.9)	407 (73.1)

History of previous/no previous CS noted, *n* (%)	Yes	31 (24.8)	61 (14.1)	92 (16.5)
	No	94 (75.2)	371 (85.9)	465 (83.5)

Year of previous CS,^b^ *n* (%)	Yes	21 (67.7)	36 (59.0)	57 (62.0)
	No	10 (32.3)	25 (41.0)	35 (38.0)

Adequate information to determine if CS interval is appropriate,^c^ *n* (%)	Yes	13 (56.5)	20 (35.1)	33 (41.3)
	No	10 (43.5)	37 (64.9)	47 (58.8)

Retroflexion/no retroflexion noted, *n* (%)	Yes	23 (18.4)	96 (22.2)	119 (21.4)
	No	102 (81.6)	336 (77.8)	438 (78.6)

NOA: nonopen access; OA: open access.

^*∗*^Restricted to 10 data elements (from 20 assessed), largely because small cell sizes required suppression of the data.

^a^Excluding 12 reports with missing data element.

^b^Only valid for the 92 who provided information on prior colonoscopy history.

^c^Only valid for the 80 who provided a valid response.
