# Supplementary figures and images for: The Quality of Colonoscopy Reporting in Usual Practice: Are Endoscopists Reporting Key Data Elements?
Source: Can J Gastroenterol Hepatol. 2016 Aug 7;2016:1929361. doi: 10.1155/2016/1929361 (PMC4992524; doi:10.1155/2016/1929361)

**Supplementary Figure 1:** Non-mandatory data elements reported by decile.

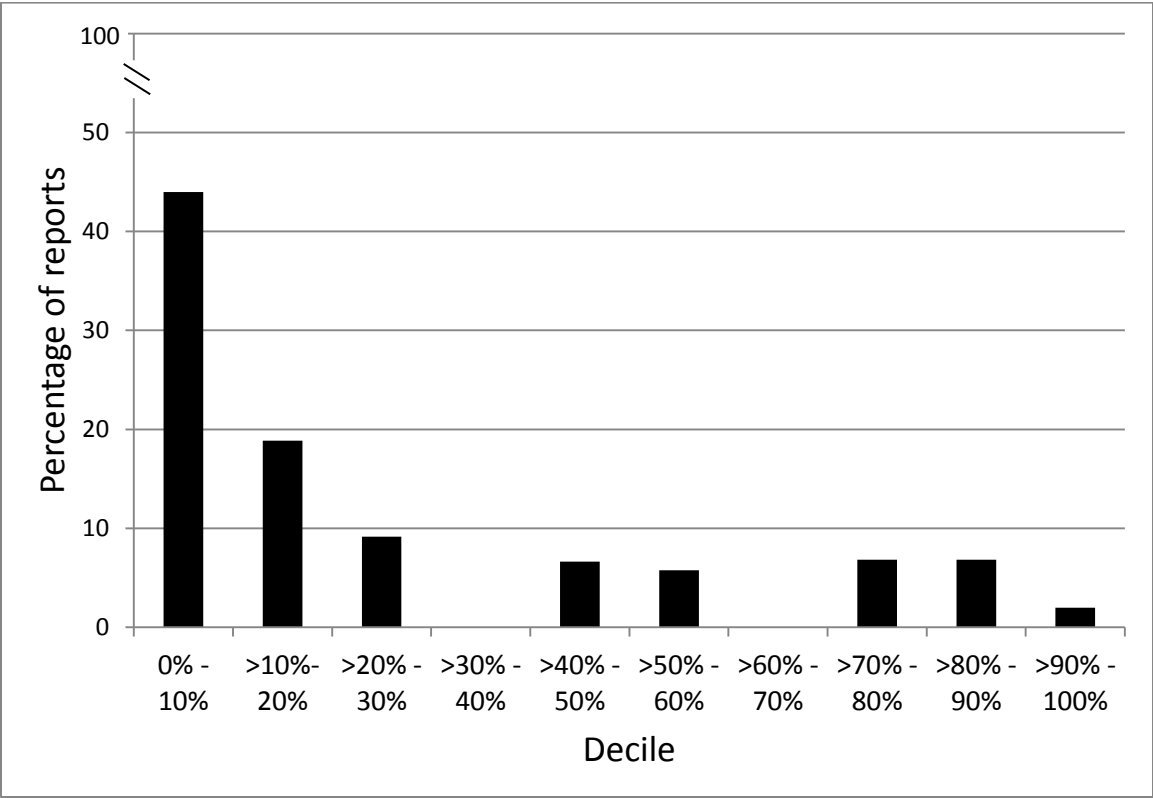

Supplement: Supplementary file 1 — Supplementary Figure 1 includes the distribution of non-mandatory data elements reported by decile. [file 1929361.f1.pdf]
